# Duodenal adenocarcinoma with skin metastasis as initial manifestation: A case report

**DOI:** 10.1515/biol-2021-0029

**Published:** 2021-04-21

**Authors:** Yixiao Fu, Cuiping Zheng, Jian Huang, Shenghao Wu, Yanyan Dai

**Affiliations:** Department of Hematology, Dingli Clinical Medical School of Wenzhou Medical University, Wenzhou 325000, Zhejiang Province, China; Department of Hematology, Wenzhou Central Hospital, Dingli Clinical Medical School of Wenzhou Medical University, No. 252, Baili East Road, Lucheng District, Wenzhou 32500, Zhejiang Province, China; Department of Pathology, Wenzhou Central Hospital, Dingli Clinical Medical School of Wenzhou Medical University, Wenzhou 325000, Zhejiang Province, China

**Keywords:** duodenal adenocarcinoma, cutaneous metastasis, case report, skin metastasis

## Abstract

**Background:**

Duodenal adenocarcinoma (DA) with skin metastasis as initial manifestation is clinically rare. In this study, we report a rare case of skin metastasis of DA.

**Case presentation:**

An 84-year-old male patient developed multiple ecchymoses on the trunk and lower extremities. Physical examination showed that the ecchymosis was dark red and had a hard texture, but showed no bulging, rupture, or tenderness. The skin biopsy implied skin metastatic adenocarcinoma. After an endoscopic duodenal biopsy, the patient was finally diagnosed with DA with skin metastasis. The patient received two courses of oral treatment of Tegafur (40 mg, bid d1–d14). However, the patient stopped taking Tegafur because of its poor effect and received Chinese medicine as a replacement treatment. Unfortunately, he was lost to follow-up.

**Conclusions:**

Early diagnosis of DA metastasis is of significant importance as prognosis of these patients is poor.

## 1 Background

Duodenal adenocarcinoma (DA) is the most common malignant type of small intestine tumor. However, it is a rare lesion that accounts for less than 0.5% of all gastrointestinal cancers [1]. In recent years, there has been an increasing incidence of this type of tumor. Because the clinical manifestations are neither obvious nor easily detectable, the rate of missed diagnosis and misdiagnosis of DA is still high. At present, there is still a lack of standardized treatment methods for this type of cancer, which translates to the poor prognosis and worse survival chances of the patients diagnosed with DA [2]. DA with skin metastases is rarely reported. In this study, we described a case of DA with skin metastasis as the initial manifestation.

## 2 Case presentation

**Figure 1:**
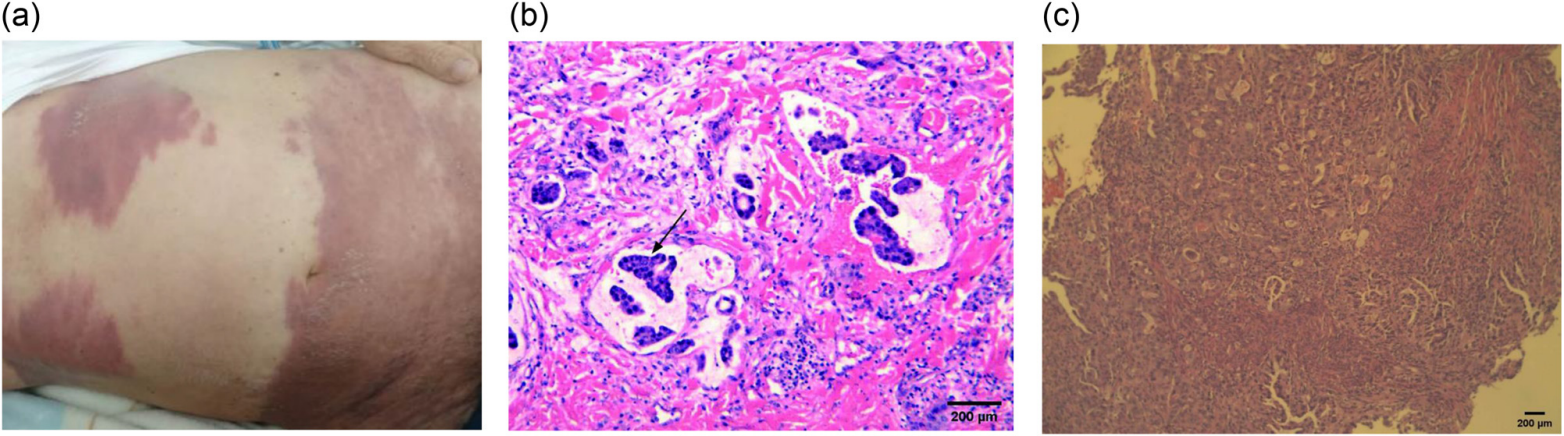
Representative images of the patient. (a) Skin metastases. (b) HE staining of skin lesion. The skin metastasis of adenocarcinoma and tumor thrombus (arrow) was found in vessels. Scale bar: 200 μm. (c) HE staining results of duodenum biopsy, showing primary adenocarcinoma of the descending duodenum. Scale bar: 200 μm.


An 84-year-old male patient was admitted to our hospital in November 2019 with skin ecchymosis. The patient developed the whole body skin ecchymosis 1 month earlier, accompanied by upper abdominal discomfort, but no abdominal pain, diarrhea, dark stool, or other discomforts such as fever. Before hospital admission, the patient experienced a rapid 4 kg weight loss. The patient was once treated for purpura simplex at a local community hospital. However, the treatment was ineffective. The physical examination results showed that the skin discoloration was mainly distributed on the trunk and lower extremities. The ecchymoses were dark red in color and had a hard texture, but showed no bulging, rupture, or tenderness. The largest ecchymosis was found on the right lower abdomen and was rectangular in shape with a size of 20 × 8 cm (Figure 1a). Laboratory examination of tumor markers showed that the carcinoembryonic antigen concentration was 3199.6 µg/L (normal 0–5 µg/L), the carbohydrate antigen 125 was 1338.3 kU/L (normal 0–35 kU/L), the carbohydrate antigen 19-9 (CA19-9) was >20220.0 kU/L (normal 0–37 kU/L), the carbohydrate antigen 153 was 58.7 kU/L (normal 0–25 kU/L), carbohydrate antigen 724 was 102.0 kU/L (normal 0–6.9 kU/L), and squamous cell carcinoma antigen was 27.06 µg/L (normal 0–2.70 µg/L). The abdominal CT and ultrasound examination of the liver, gallbladder, and spleen did not show clear space-occupying lesions. Finally, a skin biopsy and pathology analysis was performed, which suggested metastatic skin adenocarcinoma. There was a tumor thrombus in the vessel (Figure 1b). Immunohistochemistry showed a positive staining for TTF-1 CK7, CK20 weak, Villin, CEA, CDX2, CK19, and Ki-67 (60%), and negative staining for Napsin A. Based on this, we believed that the tumor was more likely to originate from the gastrointestinal tract and bile ducts. Next we performed an electronic gastroscopy, which showed chronic gastritis and mucosal lesions in the descending duodenum (Figure 1b). Pathology analysis of the duodenum biopsy samples suggested adenocarcinoma (Figure 1c). Immunohistochemical staining of the duodenum biopsy samples was positive for MLH1, PMS2, MSH2, and MSH6. The patient was diagnosed with DA with skin metastasis. Then, the patient received two courses of Tegafur (40 mg, bid d1–d14) orally. However, because of the ineffectiveness of treatment, the patient did not continue to take Tegafur and received Chinese medicine as a replacement. Eventually, the patient was lost to follow-up.

**Informed consent:** Informed consent was obtained from all the individuals included in this study.
**Ethical approval:** The research related to human use has been complied with all the relevant national regulations and institutional policies and in accordance with the tenets of the Helsinki Declaration, and has been approved by the institutional review board of Wenzhou Medical University.

## 3 Discussion

DA is a rare and aggressive gastrointestinal malignant tumor with an estimated incidence of less than 0.5 per 1,00,000 [3]. Its early onset is concealed, which may further cause delayed diagnosis and result in ineffective treatment. Skin metastasis is a relatively rare manifestation of visceral malignancies. It mostly occurs in the late stage of the disease, but it may also be an initial manifestation of potential cancer [4]. The most common skin metastasis is adenocarcinoma (60%), followed by squamous cell carcinoma (15%). Six percent of skin metastases secondary to parenchymal visceral tumors are caused by gastrointestinal tumors [5]. Skin metastasis affects 0.7–9% of all cancer patients [4]. Therefore, compared with other organs, skin is a rare metastatic disease site. Our patient was an elderly male, diagnosed with DA with skin metastasis, based on the results of skin biopsy, duodenal mucosal biopsy, and immunohistochemistry. In this case, the skin metastasis site of DA was located on the trunk and lower extremities, and the skin showed ecchymosis, rarely reported in previous studies.

DA has an early onset and is difficult to diagnose. It is often misdiagnosed as chronic gastritis, peptic ulcer, and bile duct disease. Abdominal pain or upper abdominal discomfort, jaundice, bloating, vomiting, and gastrointestinal bleeding are the most common symptoms [6]. Patients with advanced tumors and long disease duration experience rapid weight loss. At present, the diagnosis of DA mainly depends on clinical manifestations, biochemical examinations, endoscopic examination, duodenal hypotonic imaging, ultrasound examination, and abdominal enhanced CT, MRI, and PET/CT.

The CA19-9 is primarily used in the clinic as a tumor marker for colon cancer. It is a sialylated lactoyl-*N*-fucose related to Lewi plasma antigen used to diagnose pancreatic cancer with both sensitivity and specificity levels reaching 80% [7]. It is shown that the abnormal increase of CA19-9 level occurs in 36.4% of patients with DA [8]. Endoscopy is the primary auxiliary strategy for the diagnosis of DA and can be used to directly observe the location, extent, and shape of the lesion. Meanwhile, histopathological examination is helpful for tumor localization and qualitative diagnosis. Because the duodenum travels in a C shape, the technical requirements of the endoscope reaching the duodenal horizontal and ascending segments are high. Therefore, its main disadvantage is that it is not the best suit for observation of the tumors that occur in the horizontal and ascending parts. However, capsule endoscopy enables visualization of the entire small intestinal mucosa. Moreover, CT and MRI allow us to better observe the thickness of the mucosa and intestinal wall, which helps to examine the degree of tumor infiltration and the relationship with neighboring organs, to determine the possible occurrence of a distant metastasis [9].

In the present study, the most apparent clinical manifestation was the multiple ecchymoses of the skin all over the body. The ecchymoses had a hard texture, but there was no bulge and no rupture on the surface, as well as no tenderness. The level of a tumor marker CA19-9 was increased, suggesting the possibility of cancer. Through clinical manifestations and various auxiliary examinations, especially skin biopsy and endoscopy, the patient was finally diagnosed with DA with skin metastasis. This points to the conclusion that when a patient develops a skin mass, it needs to be distinguished from soft tissue tumors such as lipoma and malignant fibrous histiocytoma. Certain metastatic cancers will present infiltrating erythema, suppurative granuloma and ulcers, or rare clinical manifestations such as inflammation-like or scleroid-like masses, and erysipelas-like cancer. When skin lesions progress or expand rapidly with papules accompanied by hard texture and tenderness, with or without unhealable ulcers, it strongly suggests metastatic cancer [4]. Because skin metastasis is relatively rare compared with other sites, it is easy to become overlooked by both patients and clinicians, which may further delay the diagnosis. The clinical manifestations of skin metastases are not specific. Skin metastases caused by different solid tumors can have similar clinical manifestations and are difficult to distinguish. Therefore, we should actively perform a pathological examination to facilitate the diagnosis and promote early diagnosis and early treatment.

Because of the low prevalence of DA in the population and the limited number of clinical studies, there is no consensus on the most effective treatment strategy. At present, the preferred treatment for DA is still surgery. The most common ones are pancreaticoduodenectomy and segmental resection; however, the extent of tumor resection is still controversial [10,11,12]. Because of the lack of randomized control, the standard regimen for postoperative chemotherapy has not been defined. Chemotherapy based on fluorouracil and platinum should be considered for patients with high recurrence risk such as those with peripheral organ invasion [13]. Adjuvant chemotherapy and radiotherapy are mostly used for the comprehensive treatment of patients with advanced metastasis, and sub-targeted therapy is still in the exploration stage [14]. The 5-year survival rate of patients with advanced DA is 11.9%, and the median survival time is 2–8 months [15]. Prognostic factors such as age, weight loss, lymph node metastasis, positive margins, and TNM stage are all associated with short survival time [16]. In this study, the patient had an advanced tumor and lost the opportunity for surgery. In addition, considering the older age of the patient, Tegafur was used as a single chemotherapy. However, the patient’s advanced disease progressed rapidly, and after taking two courses of treatment, he gave up chemotherapy and changed to Chinese medicine treatment. Because of the loss of follow-up, the prognosis of this patient is unknown.

In conclusion, the onset of DA is concealed and difficult to diagnose. At the time of diagnosis, most patients are already at advanced stages of cancer. Malignant tumor with skin metastases often indicates a poor prognosis. Exploring the clinical features of skin metastasis of malignant tumors can help with the detection of tumor metastasis and improve patient’s prognosis.
